# Identification of the *Capsicum baccatum* NLR Protein CbAR9 Conferring Disease Resistance to Anthracnose

**DOI:** 10.3390/ijms222212612

**Published:** 2021-11-22

**Authors:** Seungmin Son, Soohong Kim, Kyong Sil Lee, Jun Oh, Inchan Choi, Jae Wahng Do, Jae Bok Yoon, Jungheon Han, Doil Choi, Sang Ryeol Park

**Affiliations:** 1 National Institute of Agricultural Sciences, Rural Development Administration, Jeonju 54874, Korea; linewind@korea.kr (S.S.); island1984@naver.com (S.K.); golderic@naver.com (K.S.L.); osculation@korea.kr (J.O.); inchchoi@korea.kr (I.C.); jungheon1@hanmail.net (J.H.); 2 Pepper and Breeding Institute, K-Seed Valley, Gimje 54324, Korea; wahng0@hanmail.net (J.W.D.); jaebokyoon@hanmail.net (J.B.Y.); 3 Plant Genomics and Breeding Institute, Research Institute for Agriculture and Life Sciences, Seoul National University, Seoul 08826, Korea; doil@snu.ac.kr

**Keywords:** *Capsicum baccatum*, *Colletotrichum capsici*, innate immunity, *Nicotiana benthamiana*, nucleotide-binding and leucine-rich repeat

## Abstract

Anthracnose is caused by *Colletotrichum* species and is one of the most virulent fungal diseases affecting chili pepper (*Capsicum*) yield globally. However, the noble genes conferring resistance to *Colletotrichum* species remain largely elusive. In this study, we identified *CbAR9* as the causal locus underlying the large effect quantitative trait locus *CcR9* from the anthracnose-resistant chili pepper variety PBC80. *CbAR9* encodes a nucleotide-binding and leucine-rich repeat (NLR) protein related to defense-associated NLRs in several other plant species. *CbAR9* transcript levels were induced dramatically after *Colletotrichum capsici* infection. To explore the biological function, we generated transgenic *Nicotiana benthamiana* lines overexpressing *CbAR9*, which showed enhanced resistance to *C. capsici* relative to wild-type plants. Transcript levels of pathogenesis-related (*PR*) genes increased markedly in *CbAR9*-overexpressing *N. benthamiana* plants. Moreover, resistance to anthracnose and transcript levels of *PR1* and *PR2* were markedly reduced in *CbAR9*-silenced chili pepper fruits after *C. capsici* infection. Our results revealed that CbAR9 contributes to innate immunity against *C. capsici*.

## 1. Introduction

Chili pepper (*Capsicum*) is an economically important crop providing food, spices, and medicinals [1,2,3]. Pepper is a member of the Solanaceae family, which also includes potato (*Solanum tuberosum*), tomato (*Solanum lycopersicum*), tobacco (*Nicotiana* sp.), and petunia (*Petunia* × *atkinsiana*) [4]. Members of the Solanaceae have similar numbers of chromosomes (*n* = 12), although their genome sizes can vary largely [5]. Indeed, the genomes of pepper species are typically larger, being about four times that of tomato, due to a greater fraction of repetitive sequences [6]. Of the approximately 40 species in the *Capsicum* genus [7], the only domesticated ones are bell pepper (*C. annuum*), cayenne pepper (*C. frutescens*), habanero pepper (*C. chinense*), locoto pepper (*C. baccatum*), and rocoto pepper (*C. pubescens*) [8]. Of those, *C. chinense* and *C. baccatum* harbor valuable genes for plant breeding [9]. In 1998, the World Vegetable Center identified three anthracnose-resistant varieties in these species—*C. chinense* variety ‘PBC932’ and *C. baccatum* varieties ‘PBC80’ and ‘PBC81’ [10]—paving the way for botanists to introduce anthracnose resistance into other varieties using conventional breeding and embryo rescue [11]. In particular, the *C. baccatum* resistant variety PBC80 showed broad-spectrum resistance (BSR), conferring resistance to at least two pathogenic species or to the majority of races/strains of the same pathogen [12], as well as to the three main *Colletotrichum* species causing pepper anthracnose: *C. acutatum*, *C. capsici*, and *C.* *gloeosporioides* [13,14,15]. The resistance genes originating from variety PBC80 have therefore been the focus of much attention toward their identification and characterization.

Plants and animals alike use nucleotide-binding and leucine-rich repeat (NLR) proteins to respond to invading pathogens through the activation of innate immunity [16,17,18]. The NLR family is one of the most variable gene families in plant genomes [19], and plant NLRs play various roles such as sensors, helpers, and executors in response to pathogen infection [20]. Plant NLRs are divided into three main groups based on the accompanying functional domains at their N termini: Toll/interleukin-1 receptor (TIR) domain, coiled-coil (CC) domain, and resistance to powdery mildew 8 (RPW8)-type CC (CC_R_) domain [21]. Plant NLRs also contain a central nucleotide-binding site (NBS) and C-terminal leucine-rich repeats (LRRs) [22].

Plants possess two major innate immunity systems: pathogen-associated molecular pattern (PAMP)-triggered immunity (PTI) and effector-triggered immunity (ETI) [23]. PTI signaling is initiated by the recognition of PAMPs by pattern recognition receptors (PRRs) residing in the plasma membrane, and it results in transcriptional reprograming and generation of defense components that increase resistance to a wide range of pathogens [24]. To overcome this defense mechanism, pathogens produce effectors required for inhibition of PTI and activation of effector-triggered susceptibility (ETS) [25]. Counteracting pathogen effector strategy, plants activate ETI through perception of effectors by NLRs, a well-characterized representative intracellular resistance (R) protein that recognizes specific pathogen effectors [26,27,28]. NLR-mediated ETI leads to a hypersensitive response (HR), which is characterized by a burst of reactive oxygen species and programmed cell death associated with disease resistance at the infected site [29,30,31]. HR also induces a secondary resistance response known as systemic acquired resistance (SAR), which confers long-lasting protection against a broad spectrum of pathogens throughout the infected plant [32].

Some of the sensor NLR (sNLR) receptors, such as RRS1 and RGA5, contain an integrated decoy domain that binds directly to effector [33]. However, since most characterized plant NLRs contain no integrated decoy domain [34], sNLR receptors have been proposed to interact with effectors indirectly through either an accessory protein, as in the guard model hypothesis, or via a structural mimic as in the decoy model [35,36,37]. Moreover, a subset of activated sNLRs also requires a helper NLR (hNLR) to transduce the ETI signal to downstream signaling components, with hNLRs in fact having been recently proposed to act as signaling hubs for a diverse array of sNLRs [33,38,39]. However, the noble *NLR* genes conferring resistance to *Colletotrichum* species remain largely elusive.

Pepper production is currently under threat by anthracnose disease, which is caused by *Colletotrichum* species and can result in losses of up to 80% of the harvest [40]. Therefore, identifying genes conferring resistance to *Colletotrichum* species is a high-priority goal for pepper breeding. Previous genomic studies showed that the *NLR* gene family is highly expanded and diversified in chili pepper [6,41]. Moreover, we previously mapped quantitative trait loci (QTLs) for resistance against *C. capsici* on chromosome 9, and for resistance to *C. acutatum* on chromosome 12, using composite interval mapping (CIM) [42]. Recently, we identified CbCN conferring resistance to *C. acutatum* from QTL located on chromosome 12 [43]. However, the noble genes involved in innate immunity against *C. capsici* remain to elucidated. 

We previously showed that the major *CcR9* QTL for resistance against *C. capsici* maps between the markers HpmsE143 and EtgaMccg10 on chromosome 9; *CcR9* alone explained >50% of the standing phenotypic variance for several anthracnose resistance-associated parameters [42]. Since NLR proteins play critical roles in innate immunity, here, we identified the *NLR* gene *CbAR9* from PBC80 as responsible for the QTL on chromosome 9. Molecular and genetic studies revealed that *CbAR9* confers resistance to *C. capsici* in chili pepper.

## 2. Results

### 2.1. CbAR9 Encoding a Typical NLR Protein Is Identified from the CcR9 QTL of C. baccatum PBC80

**Figure 1:**
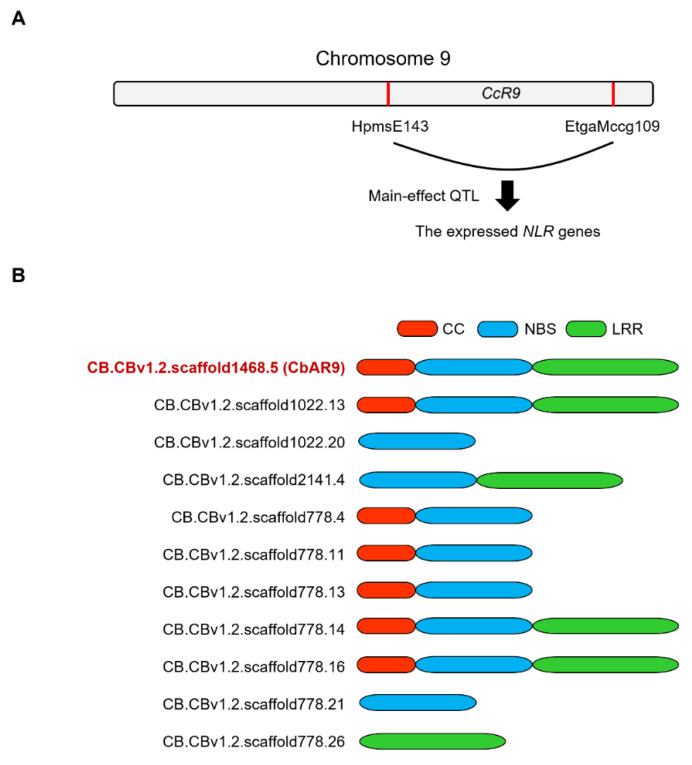
NLR-encoding candidate genes identified from the main-effector QTL of *Capsicum baccatum* resistant variety PBC80. (**A**) Schematic diagram of the identification of *CbAR9* as the main-effect QTL *CcR9* on chromosome 9 of the *C. baccatum* resistant variety PBC80. (**B**) Schematic representation of the identified candidate NLR proteins identified from the main-effector QTL of *C. baccatum* resistant variety PBC80. Red, CC domain; blue, NBS domain; green, LRR domain.

To identify candidate genes associated with anthracnose resistance, we analyzed the *CcR9* mapping interval in the *C. baccatum* genome and identified the expressed *NLR* genes (Figure 1A). The four genes such as CB.CBv1.2.scaffold1468.5/1022.13/778.14/778.16 encode a typical NLR protein, while the seven genes such as CB.CBv1.2.scaffold1022.20/21-41.4/778.4/778.11/778.13/778.21/778.26 encoded an atypical NLR proteins (Figure 1B). To know genetic divergence, we performed phylogenic analysis based on amino acid sequence of the candidate genes (Table S1). As a result, phylogenic tree showed that CbAR9 (CB.CBv1.2.scaffold1468.5) is remarkable different to the other candidate proteins (Figure S1).

### 2.2. CbAR9 Is Highly Conserved in Other Pepper Species, and the Transcription Level of It Is Dramatically Upregulated by C. capsici

**Figure 2:**
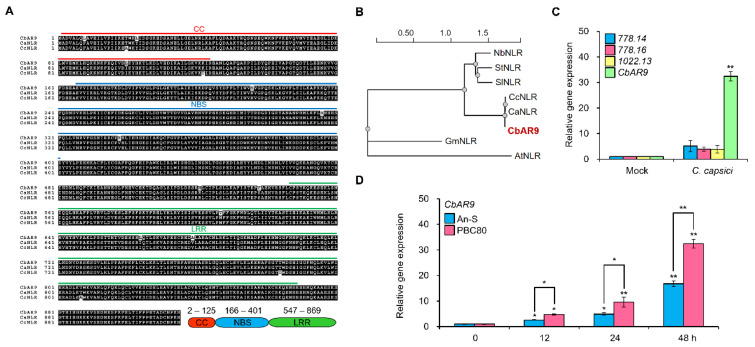
*CbAR9* encodes a highly conserved NLR protein, and its expression is induced by *Colletotrichum capsici* infection. (**A**) Multiple protein sequence alignment between CbAR9 and two homologous NLR proteins from *Capsicum annuum* (XP_016566298) and *Capsicum chinense* (PHU21239). Red, CC domain; blue, NBS domain; green, LRR domain. (**B**) Phylogenetic analysis of CbAR9. A neighbor-joining tree was constructed with NGPhylogeny software using the full-length protein sequence of CbAR9, CaNLR (XP_016566298), CcNLR (PHU21239), NbNLR (QER78241), SlNLR (XP_010319428), StNLR (XP_015161267), GmNLR (XP_006601748), and AtNLR (NP_001332515). The scale bar represents the proportion of site changes along each branch. (**C**) Relative transcription levels of CB.CBv1.2.scaffold778.14/778.16/1022.13 and *CbAR9* in PBC80 after *C. capsici* inoculation for 48 h, as determined by RT-qPCR. *Actin* served as internal reference. Data are shown as means ± SD. Asterisks indicate statistically significant differences from controls (** *p* < 0.01). (**D**) Relative *CbAR9* transcript levels in An-S and PBC80 after *C. capsici* infection for the indicated times, as determined by RT-qPCR. *Actin* served as the internal reference. Data are shown as means ± SDs. Asterisks indicate statistically significant differences from controls (* *p* < 0.05 and ** *p* < 0.01). The experiments were repeated at least three times, with similar results.

*CbAR9* encoded a typical CC-type NLR protein in the chili pepper resistant variety PBC80 (Figure 2A). The cDNA sequence of *CbAR9* consisted of 2748 bp and encoded a protein of 915 amino acids with a predicted molecular weight of 104.3 kDa and an isoelectric point of 7.06. An analysis of conserved domains with InterPro and other classification software tools revealed a CC domain from amino acids 2 to 125, an NBS domain from amino acids 166 to 401, and a LRR domain from amino acids 547 to 869 (Figure 2A). Using CbAR9 as a query, we identified two NLR proteins: one in bell pepper (CaNLR, XP_016566298) and one in habanero pepper (CcNLR, PHU21239) that were over 98% identical to CbAR9 (Figure 2A). In addition, we searched for NLR proteins with the same domain arrangement as CbAR9 in other plant species using SmartBLAST, yielding NLR proteins from soybean (*Glycine max*) and Arabidopsis (*Arabidopsis thaliana*) that are highly similar with CbAR9 over the length of the NLR domains (Figure S2). Phylogenic analysis indicated that CbAR9 clusters with other pepper NLR proteins, followed by Solanaceae proteins, which will be informative for pepper breeding using CbAR9 based on sequence similarity (Figure 2B).

Since *NLR* genes related to innate immunity are commonly induced by pathogen infection [44], we measured transcription levels of the four candidate genes encoding a typical NLR in response to anthracnose disease by infecting plants with *C. capsici*. Indeed, *CbAR9* expression was dramatically induced in PBC80 48 h after *C. capsici* inoculation. In order to monitor the expression pattern of *CbAR9* in detail, the chili pepper variety An-S and PBC80 were inoculated with *C. capsici*. *CbAR9* transcript levels rose to higher levels in the resistant variety PBC80 than in the susceptible variety An-S by *C. capsici*. (Figure 2D). Therefore, we focused primarily on *CbAR9* among the candidate genes.

### 2.3. Overexpression of CbAR9 Enhances Resistance to C. capsici Infection

**Figure 3:**
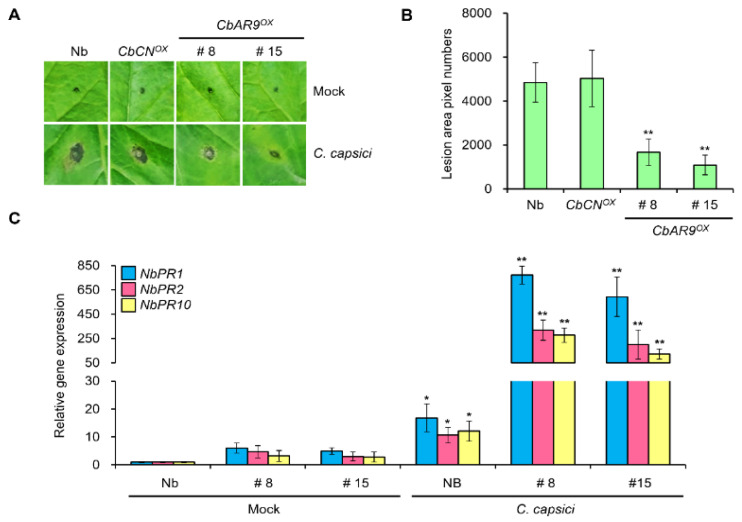
Overexpression of *CbAR9* enhances resistance to anthracnose disease caused by *Colletotrichum capsici* infection. (**A**,**B**) Anthracnose disease resistance assays of *CbAR9^OX^ Nicotiana benthamiana* plants. 4-week-old *CbAR9^OX^* and wild-type plants were inoculated with *C. capsici*. Images were captured after 6 days (**A**), and the progression of the disease was quantified using the image-based plant disease phenotyping method (**B**). Scale bars: 1 cm. Data are shown as means ± SD. Asterisks indicate statistical difference from controls (* *p* < 0.05). (**C**) Relative *NbPR* transcript levels in *CbAR9^OX^ N. benthamiana* plants after *C. capsici* inoculation, as determined by RT-qPCR. *NbActin* served as internal reference. Data are shown as means ± SD. Asterisks indicate statistically significant differences from controls (* *p* < 0.05 and ** *p* < 0.01). The experiments were repeated at least three times, with similar results.

To elucidate the biological function of CbAR9 in plants, we generated transgenic *Nicotiana benthamiana* plants constitutively expressing *CbAR9* (*CbAR9^OX^*). We validated the presence and expression of the transgene by RT-qPCR analysis (Figure S3A). Since activation of innate immunity system effects plant growth and development, overexpression of immune genes frequently causes a constitutive immunity phenotype. However, *CbAR9^OX^* did not show any visible growth phenotypes (Figure S3B). When we examined whether CbAR9 modulates disease resistance to anthracnose, surprisingly, *CbAR9^OX^* plants exhibited strong resistance to anthracnose caused by *C. capsici* compared with the wild-type plant (Figure 3A). Quantitative analysis also showed *CbAR9^OX^* plants developed smaller lesions compared to wild-type plants after *C. capsici* infection (Figure 3B). To monitor the expression of innate immunity-related genes, we performed RT-qPCR 6 days after *C. capsici* infection in wild-type and *CbAR9^OX^* plants. We observed strong induction (20- to 50-fold relative to the wild type) of the expression of *PR* genes such as *NbPR1*, *NbPR2*, and *NbPR10* in *CbAR9^OX^* (Figure 3C). However, expression levels of them did not change dramatically in *CbAR9^OX^* compared with wild-type plant after mock treatment (Figure 3C).

### 2.4. CbAR9-Silenced Pepper Plants Are More Susceptible to C. capsici and C. acutatum Infection

**Figure 4:**
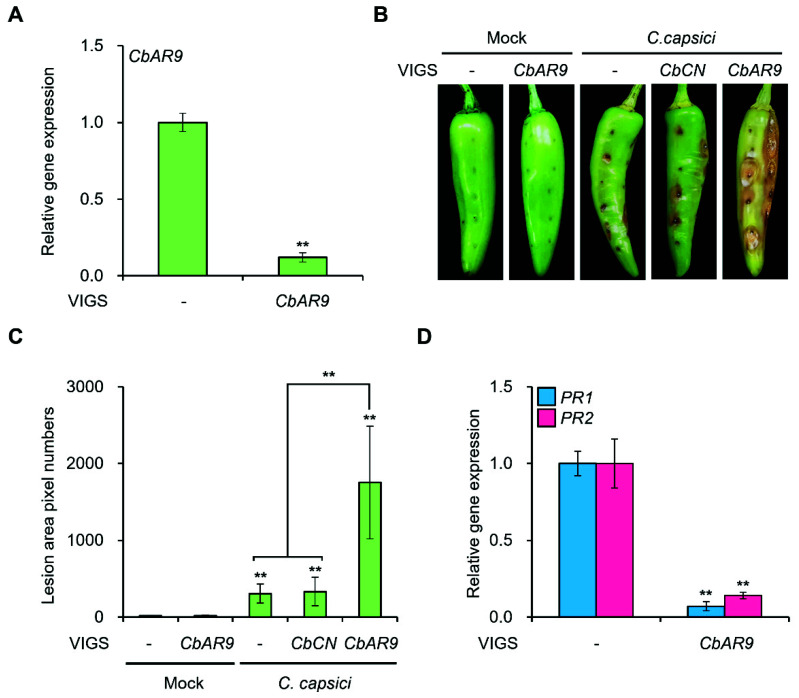
*CbAR9*-silenced chili pepper fruits are more susceptible to anthracnose after *Colletotrichum capsici* infection. (**A**) Relative transcript levels of *CbAR9* in control chili pepper fruits and *CbAR9*-silenced fruits for the PBC80 infected with *C. capsici*. Relative *CbAR9* transcript levels were determined by RT-qPCR with *Actin* as an internal reference. Data are shown as means ± SD. Asterisks indicate statistically significant differences from controls (** *p* < 0.01). The experiments were repeated at least three times, with similar results. (**B**,**C**) Anthracnose disease resistance assays with wild-type and VIGS-mediated *CbAR9*-silenced chili pepper fruits after *C. capsici* infection. *CbAR9*-silenced and wild-type chili pepper fruits were inoculated with *C. capsici*. Images were taken 6 days later, and the extent of the disease area was quantified using the image-based plant disease phenotyping method. Data are shown as means ± SD. Asterisks indicate statistically significant differences from controls (** *p* < 0.01). (**D**) Relative *PR1* and *PR2* transcript levels in *CbAR9*-silenced and wild-type chili pepper fruits after *C. capsici* infection. *Actin* served as the internal reference. Data are shown as means ± SD. Asterisks indicate statistically significant differences from controls (** *p* < 0.01). The experiments were repeated at least three times, with similar results.

To further explore the role of CbAR9 in resistance against *C. capsici*, we infected the fruits of *CbAR9*-silenced chili pepper plants with *C. capsici* for 6 days before scoring the progression of the disease. Accordingly, we silenced *CbAR9* in chili pepper fruits by VIGS, which we confirmed by RT-qPCR analysis of silenced chili pepper fruits (Figure 4A). As a result, we found that *CbAR9*-silenced pepper fruits exhibited much greater susceptibility to *C. capsici* infection (Figure 4B). A quantitative analysis of lesion size caused by *C. capsici* showed an over 5-fold increase in *CbAR9*-silenced pepper fruits compared to wild-type fruits (Figure 4C).

Since overexpression of *CbAR9* resulted in higher transcript levels for *PR* genes after *C. capsici* inoculation, we determined the effect of *CbAR9* silencing on *PR* gene expression in chili pepper by RT-qPCR on *CbAR9*-silenced chili pepper fruits inoculated with *C. capsici*. Silencing of *CbAR9* reduced *PR1* and *PR2* transcript levels in response to *C. capsici* infection (Figure 4D).

## 3. Discussion

Chili pepper is a crop with crucial economic importance worldwide, but it can also be susceptible to anthracnose caused by *Colletotrichum*. Thus, the identification of gene(s) associated with innate immunity against *Colletotrichum* species is important for pepper plant breeding. Previous genomic analyses suggested that the NLR protein family is a central regulator of anthracnose resistance in chili pepper [6,41]. However, whether, which, and how NLR proteins mediate anthracnose resistance is largely unknown. Since variety PBC80 exhibits BSR to a range of *Colletotrichum* species such as *C. acutatum*, *C. capsici*, and *C. gloeosporioides* [15], we attempted to identify *NLR* genes contributing to innate immunity involved in anthracnose resistance in the PBC80 background (Figure 1A). Accordingly, we describe here *CbAR9,* which encodes a typical NLR protein, as the likely candidate gene underlying the previously mapped main-effect QTL *CcR9* (Figure 1B). Sequence alignment and phylogenic analysis showed that CbAR9 is highly similar to homologous proteins associated with disease resistance in various plant species (Figure 2A,B). This observation suggests that CbAR9 may be conserved across pathogen-response signaling pathways, which will be useful for plant breeding of other members of the Solanaceae family.

Genes upregulated by a specific pathogen are often expected to associate with innate immunity against the same pathogen [44,45,46]. Upregulation of *CbAR9* transcript levels by *C. capsici* infection prompted us to speculate that *CbAR9* plays a role in anthracnose resistance (Figure 2C). To elucidate the function of CbAR9, we also generated and analyzed *N. benthamiana* plants overexpressing *CbAR9* (Figure S3A). Surprisingly, despite the absence of a constitutive immunity phenotype (Figure S3B), *CbAR9^OX^* plants exhibited enhanced disease resistance to *C. capsici* compared to wild-type plants (Figure 3A,B). This result suggested that CbAR9 is activated by pathogen infection, and it provides evidence that CbAR9 is involved in ETI signaling pathway triggered by the recognition of pathogen effectors. However, whether CbAR9 acts as sNLR or hNLR must be clarified in future studies.

Since the activation of NLR proteins commonly leads to significant transcriptional reprogramming [47], we monitored the expression of genes related to innate immunity. RT-qPCR analysis showed that *PR* genes are dramatically induced in *CbAR9^OX^* plants. As a complementary analysis, we also silenced *CbAR9* by VIGS (Figure 4A). *CbAR9*-silenced chili pepper fruits showed increased susceptibility to *C. capsici* infection (Figure 4B,C), and *PR1* and *PR2* transcript levels decreased significantly compared to wild-type plants (Figure 4D). These results showed that CbAR9 is involved in disease response to *C. capsici*.

Surprisingly, although CbAR9 is 98% identical to the two homologous proteins of other *Capsicum* species lacking a resistance to *C. capsici* (Figure 2A), it dramatically increased the innate immunity against *C. capsici* (Figure 3 and Figure 4). These results imply that the 11 amino acid difference of CbAR9 may be an important site for CbAR9 activation (Figure 2A). A recent study showed that *Arabidopsis* RRS1/RPS4 NLR protein immune receptor complex is regulated by phosphorylation [48]. Therefore, Ser 216 and Thr 607 on CbAR9 especially need to be tested in future. Investigating and understanding the basis of genetic resistance to relatively understudied pathogen species such as *Colletotrichum* is important. The effectors from *Colletotrichum* species and their dedicated NLR receptors in chili pepper remain unknown. Thus, the identification and a deeper understanding of *NLR* genes from variety PBC80, which exhibits disease resistance to *Colletotrichum* species, are important for plant breeding. Our findings will offer new options for pepper plant breeding.

## 4. Conclusions

Here, we identified *CbAR9* encoding nucleotide-binding and leucine-rich repeat protein as the causal locus underlying the large effect quantitative trait locus *CcR9* from the anthracnose-resistant chili pepper (*C. baccatum*) variety PBC80. Interestingly, despite the absence of a constitutive immunity phenotype, the anthracnose resistance and transcript levels of *PR* genes were significantly increased in *CbAR9*-expressing transgenic *N. benthamiana* lines after *C. capsici* inoculation. Conversely, resistance to anthracnose and transcript levels of *PR* genes were markedly reduced in *CbAR9*-silenced chili pepper fruits after *C. capsici* infection. Taken together, we revealed that *CbAR9* which is located on *CcR9* locus of PBC80 contributes to innate immunity against *C. capsici* and regulates gene expression involved in disease response.

The *C*. *baccatum* resistant variety PBC80 contains the noble genes conferring disease resistance and BSR to *Colletotrichum* species, and they are proposed as a vital biotechnological target for introducing anthracnose resistance. Therefore, identification of CbAR9, a positive regulator of innate immunity to anthracnose without a constitutive immune phenotype, may offer new possibilities for crop improvement. Moreover, further elucidation of CbAR9 will explain plant defense mechanism against *Colletotrichum* species and the role of NLR related to its presence in pepper.

## 5. Materials and Methods

### 5.1. Plant Material and Growth Conditions

Seeds of chili pepper (*Capsicum baccatum*) variety ‘PBC80’ and *Nicotiana benthamiana* (*N. benthamiana*) were surface sterilized in 70% ethanol for 1 min, followed by 5% sodium hypochlorite for 3 min, and then washed thoroughly with sterilized distilled water. Clean seeds were then sown on half-strength Murashige and Skoog (MS) medium and placed in a chamber with a programmed photoperiod at 28 °C. Depending on the experiment, aseptic seedlings were kept in a growth chamber or transplanted into pots containing soil in a greenhouse under a programmed 16-h light/8-h dark photoperiod at 28 °C. The humidity was adjusted to 50%.

### 5.2. Cloning of CbAR9 and Web-Based Analysis

The full-length cDNA of *CbAR9* (PHT39462) was amplified by PCR with the primers listed in Table S2 from first-strand cDNA prepared from total RNA extracted from variety PBC80. The *CbAR9* cDNA was inserted into the pENTR entry vector using Gateway BP Clonase II enzyme (Invitrogen, Waltham, MA, USA), and then recombined into destination vectors [49] using Gateway LR Clonase II enzyme (Invitrogen, Waltham, MA, USA) as per the manufacturer’s instructions. For subcellular localization and generation of transgenic plants, the *CbAR9* cDNA was recombined into the pEarleyGate101 and the pEarleyGate201 vectors, respectively.

The phylogenic tree was generated using the NGPhylogeny (https://ngphylogeny.fr/workflo-ws/oneclick/, accessed on 16 May 2020).The CbAR9 amino acid sequence was analyzed for predicted molecular weight and isoelectric point using IPC (http://isoelectric.org/, accessed on 21 July 2020) tools. Conserved domains were predicted with the InterPro software tool (https://www.ebi.ac.uk/interpro/, accessed on 16 May 2020). SmartBLAST (https://blast.ncbi.nlm.nih.gov/smartblast/?LINK_LOC=BlastHomeLink, accessed on 16 May 2020) was used to identify homologs in other plants.

### 5.3. Total RNA Extraction and RT-qPCR Analysis

Total RNA was extracted using TRIzol reagent (Invitrogen, Waltham, MA, USA). First-strand cDNA synthesis was initiated from 2 µg of total RNA using Superscript III reverse transcriptase (Invitrogen, Waltham, MA, USA) according to the manufacturer’s instructions. RT-qPCR was performed using gene-specific primers (Table S1) on a MyiQ Real-Time PCR System (Bio-Rad, Hercules, CA, USA) using SYBR Green Master Mix (Bio-Rad, Hercules, CA, USA) under the following conditions: 40 cycles of denaturation at 95 °C for 10 s, annealing at 58 °C for 15 s, and extension at 72 °C for 30 s. Relative transcript levels were quantified using the comparative Ct method, with *Actin* as an internal reference. All experiments were independently conducted at least three times.

### 5.4. N. benthamiana Transformation

To generate transgenic plants overexpressing *CbAR9*, the pEarleyGate201-CbAR9 construct was introduced into Agrobacterium strain LBA4404 by electroporation. Positive colonies harboring the construct were selected based on resistance to appropriate antibiotics and grown in YEP medium. Agrobacterium-mediated leaf disk transformation was performed as previously described [50]. Transgenic *N. benthamiana* lines were selected on MS plates containing antibiotic and propagated to obtain homozygous T_3_ lines. All transgenic lines were verified by RT-qPCR, and two T_3_ lines were chosen for detailed analyses.

### 5.5. Anthracnose Disease Resistance Assay

To test whether CbAR9 modulates disease resistance to anthracnose, we infected *CbAR9^OX^* plants with *C. capsici* and followed the progression of the disease. Previously reported *CbCN^OX^* plant lines conferring disease resistance to *C. acutatum* were used as the control [43]. *C. capsici* was cultured on potato dextrose agar (PDA) medium for 3 to 7 days with a programmed 12-h light/12-h dark photoperiod at 28 °C. Conidial suspensions were collected in 3 mL of sterile distilled water using a scalpel and filtered through two layers of cheesecloth. Their density was adjusted to 1 × 10^6^ conidia/mL by adding sterile distilled water and counting with a hemacytometer. Anthracnose disease resistance was assayed using the pinning method with a toothpick for *N. benthamiana* leaves and by microinjection for chili pepper fruits, as previously described [51]. The inoculated plant tissues were incubated at 28 °C for 6 days. For a more quantitative analysis, the extent of lesion areas was measured using an image-based plant disease phenotyping method [52]. All experiments were performed in triplicate. Consistent results were obtained, and representative data from one replicate are shown.

### 5.6. Virus-Induced Gene Silencing (VIGS) of CbAR9 in Chili Pepper

Transcription levels of *CbAR9* were silenced using virus-induced gene silencing (VIGS). Previously reported construct of VIGS for *CbCN* silencing was also used as the control [43]. VIGS was performed as previously described [53] with slight modifications. Briefly, the target sequence of *CbAR9* for VIGS was amplified by PCR using specific primers (Table S1) and inserted into the pTRV2 vector. The pTRV1 and pTRV2-CbAR9 constructs were transformed into Agrobacterium strain GV3101 by electroporation. Positive colonies harboring the constructs were selected based on resistance to appropriate antibiotics and grown overnight at 28 °C in YEP medium. The cultures were harvested by quick centrifugation and resuspended in MMA buffer (10 mM MES, 10 mM MgCl_2_, and 200 µM acetosyringone) to a final OD_600_ of 0.7. Cell suspensions harboring pTRV1 and pTRV2-CbAR9 were mixed in a 1:1 (*v*/*v*) ratio and infiltrated into chili pepper fruits. The inoculated fruits were placed in the dark at 20 °C in 50% relative humidity for 48 h. The plants were then moved to the growth chamber under a 16-h light/8-h dark photoperiod at 28 °C and incubated for 6 days.

### 5.7. Statistical Analysis

All experiments were independently conducted at least three times, and the data were analyzed by *t*-test using GraphPad Prism 8.0 software, and asterisks denote significant differences (* *p* < 0.05, ** *p* < 0.01).

## Data Availability

The data presented in this study are available in the article or the Supplementary Materials.

## Supplementary Materials

The following are available online at https://www.mdpi.com/article/10.3390/ijms222212612/s1.
