# Supplementary material for: Identification of the Capsicum baccatum NLR Protein CbAR9 Conferring Disease Resistance to Anthracnose
Source: Int J Mol Sci. 2021 Nov 22;22(22):12612. doi: 10.3390/ijms222212612 (PMC8620258; doi:10.3390/ijms222212612)
Supplement: Supplementary file 1 [file ijms-22-12612-s001.zip › ijms-1449353 - supplementary for publication.pdf]

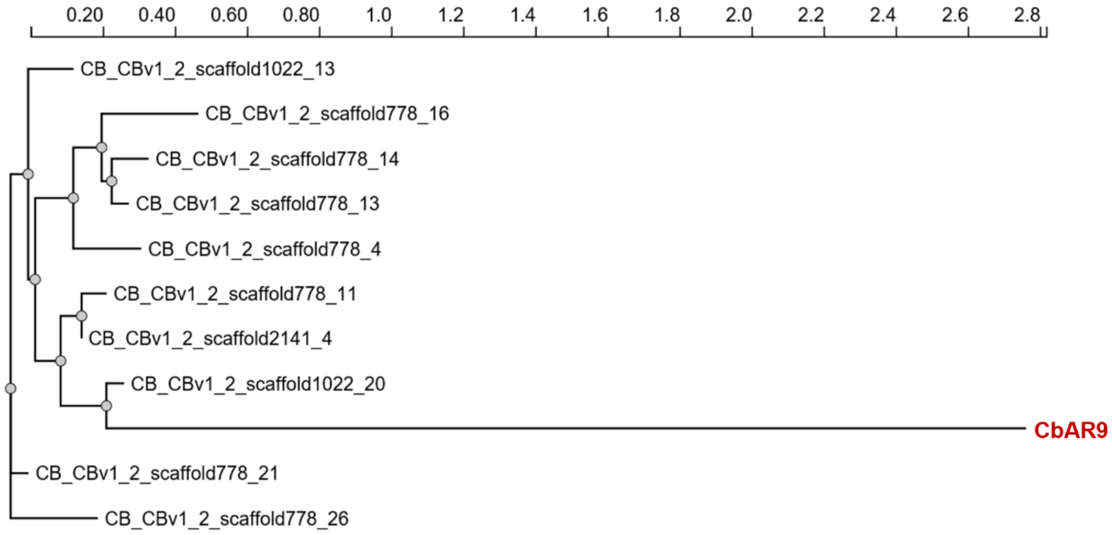

Supplementary Figure S1. A phylogenetic tree of the identified NLR proteins. A neighbor-joining tree was constructed with NGPhylogeny software using the full-length protein sequence of CbAR9 (CB.CBv1.2.scaffold1468.5) and CB.CBv1.2.scaffold1022.13/1022.20/2141.4/778.4/778.11/-778.13/778.14/778.16/778.21/778.26. The scale bar represents the proportion of site changes along each branch.

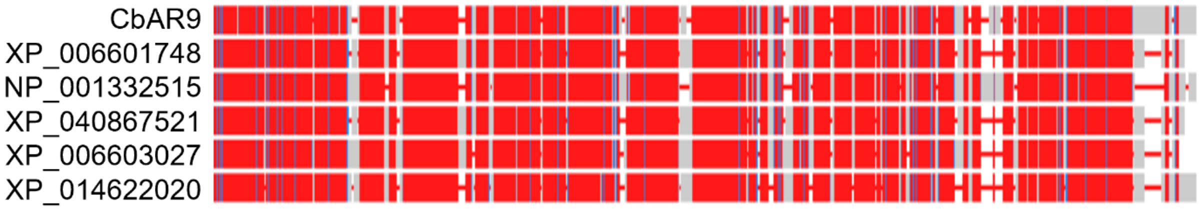

Accession	Organism	Description
XP_006601748	*Glycine max*	NBS-LRR disease-resistance protein scn3r1 isoform X2
NP_001332515	*Arabidopsis thaliana*	Disease resistance protein RPP8L3
XP_040867521	*Glycine max*	NBS-LRR disease-resistance protein scn3r1 isoform X1
XP_006603027	*Glycine max*	Disease resistance protein RPP13
XP_014622020	*Glycine max*	Disease resistance protein RPP13

Supplementary Figure S2. Identification of proteins with a similar NLR domain as CbAR9 in various plant species. The proteins were identified using the SmartBLAST program.

A

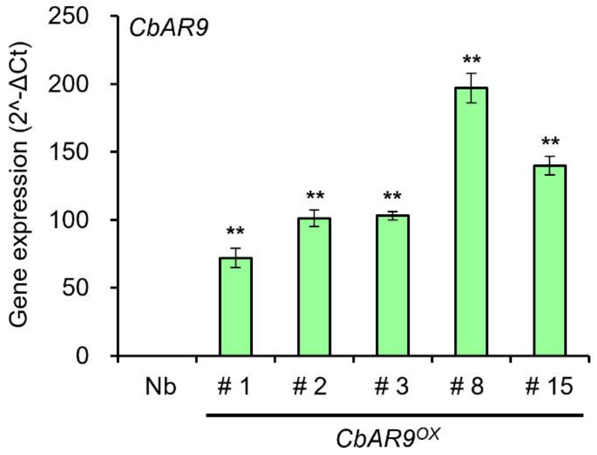

B

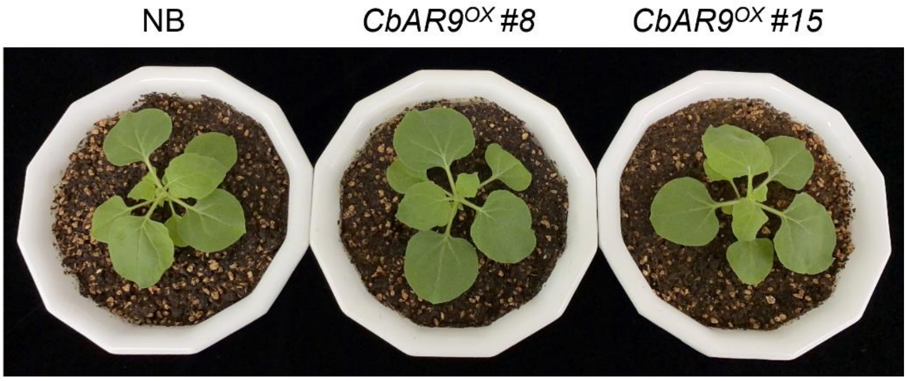

Supplementary Figure S3. Verification and phenotype of *CbAR9*-overexpressing plant lines. (A) Analysis of *CbAR9* expression in *CbAR9*^{OX} and the wild-type *Nicotiana benthamiana* plants, as determined by RT-qPCR. *NbActin* served as the internal reference. Data are shown as means ± SD. Asterisks indicate statistically significant differences from controls (** $P < 0.01$). The experiments were repeated at least three times, with similar results. (B) Growth phenotype of *CbAR9*^{OX} without biotic stress. Plants were grown in pots containing soil in a greenhouse under a programmed 16-h light/8-h dark photoperiod at 28 °C. The humidity was adjusted to 50%.

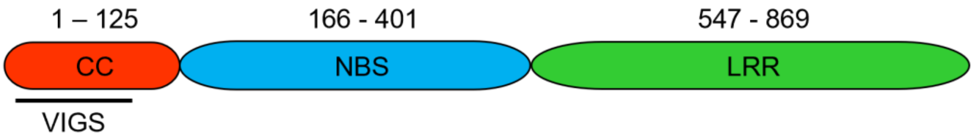

Atggcagatgtagcattacaacttgctgtggagatattggttccgataataaaggagacttggaagttgattgatagtggaaaagaagat
tctgcaaatgaactactgggagaacttaatcgtttaaaggccttcttgcaggatgctgcaaaatatcgtcaaagcaacagtgaacagtg
gaaaaactttgtcaaggaggtccaagttatggtatatgaagctgagggtctcattgataagctcatggttgagatgaagctgcaccaaaa
gaaaaataaatttgaacagatcgtggatcttaaatatcacaaaacccttaaggatcttgtagaagacatcaaagcaattcttggaaaggt
gaagaagattcgcgaagcaaatctgcaggctttccaggcaaagccaatccttgattatcagccggaaattgttgcccccggcacacag
gatactttattggaagaaaacaaagttgtcggttttgatgaggaagcaaaagtagtgatcaagcgacttgttaaaggaaccaaagattta
gatgttatccctgtggtggggttgcctggactagggaaaaccactctggcaataaaaatctctaaagatccgcaagtttcctatgatttttc
ctgaccatttgggtgagcgtaggcccgcaatccaaactgaagggggtcttccttagtattctgaaagcgttcaagaaacagactacaga
atatcaagacatggatgtgaaggaattgccaaagataatatgtgaattcattgacaaaggcggcaaatgtctcattgtattggatgacgt
gtggacaacagacgttgtggatgctgtcatgaatgttttccccgaaaaaagcaaaggccaccgtatcatgatcaccactcgtgatggac
gtattggtagatatgccaatgctaatcctcacatgttgaaatttctggaaatggaggaaagtttccaattgttggtaaatagagtttttggcag
taatattcgaaggtgtcctgaagagttaatagaacatggggaaagcatcgcaaaacaatgttttggagtgccacttgcagttgtggtaatt
gcaggagctctaagaggacgcaccagcaaaagtgactggaaaatggttgaggacaatgtaaaacaccttataaataaagatgatga
cccaaaaagctgcttgaaatttgtggaaatgagttatgtttatctacccgaagagatgaaggcgtgtttcctatattgtggtgcctttccaca
aggctttgaaattcctgcttggaagttgatccgcttatggatttccgagggattgataaactccaacttaacaggaagccccgaggatata
gcagagtattacttgaacgaccttatcaacaggaacttagtaatagtagtgcagaaaagggctaatggtcaagtaaaaacatgccgtat
tcatgacatgttacaccagttttgcaagatagaggctaataacgaaggtcttttccacgaagtatgtgaaaaaacagatcaggctggtctt
tctataccagatctagatagttcgcgtcacttgtgtattccactctctcttttgaaagcttttatctccaccgaaccatatgctgagcatgttagat
cattcttgtgtttttccacaaaacaaaaagaaagtgagaagcttagcaacattcaacaactccacaaagcctttccactggtcagggtctt
ggatgttgaatcccttgaatttagtttcagcaagtatttcagagagctatatcatttgaggtacatttccatctcagttgaaagcagtgtccttcc
gacgttcttcggtaaattttggaatttacaaactcttataatttatacaaaggcatccaccattgaaattaaagctgaaatatggaacatgct
acggttgaggcatgtgcacaccaatgtccctgcaaaattgccatcctctactacccacacagtagatgaatcttctcgcctacaaactttg
tctaaagttgcaccagaaagttgcagagaagatgcgcttgcgcgggcttgtaatctcagaaaactaactattcaagggaaaatggctg
attttcttgaaattaacatgggtgggttcaacaactttcaaaagctaaagtgcctggagcaattgaaactgctgaatgataatgtagacag
atccatgagcggagttcttcaccttcctcccgcattccccaaatttctatgcaaactgaagaagttaactttgtcaaatacaaggtttgcttgg
agcgaggcgagtagattggggcagttggaatgccttgaggtcctaaagctaaaagaaaatgcatttagtgggacaacatgggattca
gagattggaggttttaaccaactaaaggtattatggattgaaagggcagaccttaaaacttggaaggtcgcaaatcttcaatttcaaaga
cttcagtgtcttgttgttaaatcctgcgatgagcttgaggctgtaccaattgagttggctgatgtacgtacccttcaagaaatgacgctggag
cacacaaaaaaggctatcaaatctgcgaatgctatcaaatgcaggaagcaagagatgcatcgtgagcacatgcagaaagctgcag
aaagcaaatctggaacagaaatcgaaggcaagaaaaaggtgagtgaagatatggagagtttcaaattcgagctcactatattccccc
ctgaaacggctgattgcaaccccaaacattga

Supplementary Figure S4. The target region of *CbAR9* for VIGS. The cDNA sequence of *CbAR9*. Yellow fluorescent sequence indicates the target region of VIGS. Red, CC domain; blue, NBS domain; green, LRR

CB.CBv1.2. Scaffold	Amino acid sequence
1468.5<br>(CbAR9)	MADVALQLAVEILVPIIKETWKLIDSGKEDSANELLGELNRLKAFLQDAAKYR<br>QSNSEQWKNFVKEVQVMVYEAEGLIDKLMVEMKLHQKKNKFEQIVDLKYHKTL<br>KDLVEDIKAILGKVKKIREANLQAFQAKPILDYQPEIVAPGTQDTLLEENKVV<br>GFDEEAKVVIKRLVKGTKDLDVIPVVGLPGLGKTTLAIKISKDPQVSYDFFLT<br>IWVSVGPQSKLKGVFLSILKAFKKQTTEYQDMDVKELPKIICEFIDKGGKCLI<br>VLDDVWTTDVVDAVMNVFPEKSKGHRIMITTRDGRIGRYANANPHMLKFLEME<br>ESFQLLVNRVFGSNIRRCPEELIEHGESIAKQCFGVPLAVVVIAGALRGRTSK<br>SDWKMVEDNVKHLINKDDDPKSCLKFVEMSYVYLPEEMKACFLYCGAFPQGFE<br>IPAWKLIRLWISEGLINSNLTGSPEDIAEYYLNDLINRNLVIVVQKRANGQVK<br>TCRIHDMLHQFCKIEANNEGLFHEVCEKTDQAGLSIPDLDSSRHLCIPLSLLK<br>AFISTEPYAEHVRSFLCFSTKQKESEKLSNIQQLHKAFPLVRVLDVESLEFSF<br>SKYFRELYHLRYISISVESSVLPTFFGKFWNLQTLIIYTKASTIEIKAEIWNM<br>LRLRHVHTNVPAKLPSSTTHTVDESSRLQTLSKVAPESCREDALARACNLRKL<br>TIQGKMADFLEINMGGFNNFQKLKCLEQLKLLNDNVDRSMSGVLHLPPAFPKF<br>LCKLKKLTLSNTRFAWSEASRLGQLECLEVLKLKENAFSGTTWDSEIGGFNQL<br>KVLWIERADLKTWKVANLQFQRLQCLVVKSCDELEAVPIELADVRTLQEMTLE<br>HTKKAIKSANAIKCRKQEMHREHMQKAAESKSGTEIEGKKKVSEDMESFKFEL<br>TIFPPETADCNPKH
1022.13	MLSFFANLCIALFVIPLTSCNPAVDMAHSSVASLMRTIESLLTSNSPMQTLIC<br>GHRDEICPLHEKISSLEVFLKNFEKNNVGELTDLEVQIKEVANIVEQTIQLRV<br>TEVLLANDENLREKSHERLSDSLQQVAEDIDRIWKESTNIQDKGKHASKESTV<br>QEFLSSSRNILNVENSMVGRDDQRKRLVEDLTRSYSGEPKVIAIVGMGGIGKT<br>TLANAVYNDACIRSHFDVCAWATISQQHNVKEILLSLLRSTKSDSFDMNDEAE<br>LANMLQKSLKGKRYLIVLDDMWKTESWDAVRLCFPSENKGSGILLTTRNSEVA<br>RDAGTENLSLQMDLMGPDESWNLFKSVAFANEALPSEFETIGKQIVEKCHGLP<br>LTIAVVARLLKSKRAIEEWENVAKDVKSFVTNDPDERCSRVLRLSYNHLTSDL<br>KTCLLHFGIFPEDSEIPVKNLMRSWMAEGFLKLENDLEGEAVKCLQELVDRCL<br>VLVCKKSLDGTKIRSCKVHDLIYDLCLREIERDNIFIMNDIVLEFDSGWTYLS<br>LRKMKPFKRVTVDEIDYCRSGLYRALLTPVHRQLRDHDNNDLFNRTRSIFSSY<br>PCTSHLFRKSELIHFKLLKVLELRNIKMDHFPVQILSLIWLRYLLLYFRKYLE<br>IPPEICRLWNLQTFIVQGPSLWKITFPEEILGLMQLRHLKLPEFYLPNPPSVS<br>ADKGSHVSFSNIQTISSMSPCCCTKEFIMRIQNVKELGLSAYQIDSSDGPLNS<br>LVHLQQLETLSLTYRFGGFWPASAKAFPATLKKLKLDKTYLSWSYLDIIAELP<br>NLEVLKLMYRACDGEEWRPTVMGFNRLKLLLIDDNDLKYWKATDDDFPVLERL<br>VLRRCHYLEKIPIEFAGIHSLQLIELNWGPPKLGDSAARIQQEQEEIGNNPV
1022.20	MGGIGKTTLANEVYNDACIRSHFDVCAWATVSQQHNVKEILLSLLRSTKGDTF<br>DMNDEAELANMLQKSLKGKRYLIVLDDMWKSEAWDAVRLCFPSENKGSGILLT<br>TRNTDVALDAGTKNLSLQMDLMGLDESWNLFKSVAFANEALPSEFETIGKQIA<br>DECHGLPLTIAVVAGLLKSKRAIENWKSVEEDVKSFVSNDPDERCSRVVGLSY<br>DHLTSDLKACLLHFAIFPEGSEIPVKNLRRSWMAEGFLKLENDLEGEAEKCLQ<br>ELVDRCLVLVCKKSLDGTKIRSCKVHDLIYDLCLREIQRGNVFIMNDIVLYVA<br>EPECLDLSMHKMQPFKRVPGDLIDNPYFGLYRALLTPVHRQLRDHDNNDLLKQ<br>TCSIFSFHLNDSCFILKSELIHFKLLKVLVLRHRDIDNFPLQILSLIWLRYLS<br>LVVHGHLLIPPEICRLWNLQTLVVKEYDPSFVTCPVQIWELVQLRHLKLPRFY<br>LPDRPSVSVDKERDVGFSNLQTTSRLSSCCCTREVIMGIQNVKKLEISGDEDD<br>YESFWESGLVNNLVHLHQLETLSLTGTYILWPRTLPASTPSAKSFPATLKKLN<br>LTSTYLSWSYLDIIAELPNLEVLKLMGFACCGEGEEWHPIVMGFNRLKILLIE<br>RSFLKYWKATDDNFPVLERLVLINCKCLKEIPIEFAEIHSLQLIEVALPYKSN<br>IPWAIWNPPLTQRGSPQPECAARVGVSDSDTPHGSRPA

CB.CBv1.2. Scaffold	Amino acid sequence
2141.4	MQNVENSMVGRDDHRKWLVEDLTRSYSGEPKVIPIVGMGGIGKTTLANKVYNN<br>ACIRSHFDVCAWATVSQQHNVKEILFSLLRSTKGGTFDMNDEAELANMLQRSL<br>KGKSYLIVLDDMWKSEAWDAVRLCFPSENKGSGILLTTRNTEVARDAGTENLS<br>LQMDLMGSNECWNLFKSVAFANEALPSEFATIGKQIAEKCHGLPLTIAVVAGL<br>LKFKRAIEDWKSVAKDVKIFREDSEIPAKKLMRSWMAEGFLKLENDLEGEAEK<br>CLQELVDRCLVLICKKSLDGTKIRSCYGICRHSLFKVRGHTLFSVLVTFPGEI<br>WGLMQLRHLKLPRFNLPDCPSGSVDKGRHLDFSNLQTISYLSLDCCTKEVIMG<br>IQNVKELGICGYETDSNRILNNLVHLQQLETLSFIFCFAQFPGTLKKLKLEST<br>FLSWSYLDIITELPNLEVLKLMGFACDGEEWHPNVRGFTRLKLLLIEDYFLEY<br>WRQFSCP
778.4	MAHASVASLTGTIESLLTSNSPMQSLSCDHREEICDLHKKISSLEIFLKNFDK<br>NNVYGQITDLEVELKEVANIAEQTIQLRVTEVVLVNDEKTHERLSDTLQLVAE<br>DIDRIWKVSTKIKDKGKQVSEGSLVQDFSSSINNILNVNNHTVGRDDQKERLL<br>EHLTKSYSGESKVIPIVGMGGIGKTTLAKEVYSYESILRRFDVSAWATVSQQQ<br>NIKEILLSLLQSTIKMDDTVKMKGEAEVKSLKRKRSRILLTTRNDEVGCYAGI<br>ENISLRMSFMDQDESWNLFKSAAFSSEALPYEFETVGKQIADECHGLPLTIVV<br>VAGLLKSKRAIKDWESVAKDVKSFFTNDPDERCSRVLGLSYNHLTSDLKTCLL<br>HFGIFREDSEIPAKKLMRSWMAEGFLKLENDLEGEAEKCLQELVDRCLVLVCK<br>RSLDGTKIRSYKVHDLIYDLCLKEIQRENIFIMKDIVVWVCISECQFLRMHKM<br>QPFKCVTDDEIDYSRYGLYRALLTPVHRQLRDHDNNDLLKRTHSIFPFGLNDL<br>FFMFKSELIHFKLLKVLNLSHVRIDSFPLQILNLIWLRYLALLIYVNLKIPRE<br>ICRLWNLQTFIVKGMRLSVITFPEEIWGLMQLRHLKLSRFYLPDCPSGSVNKG<br>RHLDFSNIQTISYLSQRCCTKEVIMGIQNVKELGISGGDEIDSNGPLNNLVHL<br>QQLETLSFIFCLKILPASAKAFPATLKNLKLERTLTSWSYLDIIAEFPNLEVL<br>KLMDHACLGDEWHPIVRGFTRLKLLLIEEDNFLKHWKATDDNFPVLERLVLKK<br>CHNYKEIPIKSSSYPCTSAYSFQITQKVLDLSHVRIDSFPLQLLNLIWLRYLA<br>LLIYVNLKIPREICRLWNLQTFIVKGMRLSVITFPEEIWGLMQLRHLKLSRFY<br>LPDCPSGSVNKGRHLDFSNIQTISYLSQRCCTKEVIMGIQKVKELGISGGDEI<br>DSNGPLNNLVHLHVQEESKKIQDHNGRQESTWSLAQDKSSEKLLNLEVSNNMV<br>GRGKEKKRVLEELRGGSSDELKIIPIVGMGFIGKTTLAKQVFNVKEILLSLLQ<br>SIIQIDDKVYSRDEAELADLLQKSLKRKRYLIVMDDIWSDKAWDDMRQCFPID<br>NNRS
778.11	MAHASVASLMRTIESLLTINSPMRSLSFDHREELSALREKTSSLEVVVKNFEK<br>NNVCGEITDFEVEVKEVANFVEHTIQLQVTEVLLANDENLREKAHWWLSDSLQ<br>QVEEDIDIIWEESIMIQDEGKQASKEPTVQEFPSSSKDMQTVESSMVGRDDHR<br>KRKRLVEDLTRSYSGEPKVIPIVGMGGIGKTTLANEVYNNACIRSHFDVCAWA<br>TVSQQHNVKEILFSLLRSTKGGTFDMNDEAELANMLQRSLKGKRYLIVLDDMW<br>KNEAWDAVRLCFPSENKGSGILLTTRNTEVARDAGTENLSLQMDLMGPNECWN<br>LFKSVAFANEALPSEFETIGKQIAEKCHGLPLTIAVVAGILKSKRAIEDWKSV<br>AKDVKSLVTNDPDERCSRVLGLSYNHLTSDLKACLLHFGIFREDSEIPAKKLM<br>RSWMAEGFLKLENDLEGEAEKCLQELVDRCLVLICKKSRDGTKIRSCKVHDLI<br>YDLCLREIQRGNVFIMNDIVLDSMPVTGLFRVLLTPVHRQLRDHDNNDHLKRT<br>RSIFSCHLKYSYSILKSELIHFKLLSVLELRHRLIDNFPPVILSLIWLRYLSL<br>LCRENLDIPPEICRLWNLQTFIVQGMRTYIFPSDLVTFPEEIWGLMQLRHLKL<br>PRFNLPDCPSGSVDKGRHLDFSNLQTISYLSLDCCTKEVIMGIQNVKELGICG<br>YETDSNRILNNLVHLQQLETLSFIFCFAQFPACAKAFPAMLKKLKLESTFLSW<br>SYLDIIAELPNLEVLKLMGFACDGEEWHPNVRGFTRLKLLLIEDYFLKYWNAT<br>DDNFPVLERLVLNKCHNLKEIPIEFAEIHTLQLIELERCLPELGESAARIQKE<br>QEDLGNDPVDVRISRPCDDDGGGGGGGGDDEEYFDAVDVAEDDDDENSDVDVA<br>EDDDDEDSDVDVAKDDDDEDFDADAAEHDD

CB.CBv1.2. Scaffold	Amino acid sequence
778.13	MITPLLGYTPDLHSGSFDPNVRCSYHSDVQGHSIEDCRALRSEIERMIQDKSI<br>MVSGHNHQERNENTVDMAHASVASLMRTIESLLTINSPMRSLSFDHREELCAL<br>REKVSSLEVFVKNFEKNNVSGEMTDFEVEVKEVASASEHTFQLRVTEVLLEND<br>EKIHERLSDTLQLVAEDIDRIWKVSTKIQDKGKQASKESTVQDFSSSKNNILN<br>VNNIMVGRDDQKERLLEDLTASYSGEPKVIPIVGMGGIGKTTLAKEVYNNESV<br>LRRFDVHAWATVSQQHDRKEILLGLLRSTIKMEDRVKTRGEAELADMLQKSLK<br>RKRYLIVLDDIWSCEVWDGVRQCFPTEDNAGSRILLTTRNNEIACYAGTENLS<br>MQMDFMDQDESWNLFESAAFSSEALTXNTFPSVNRIP
778.14	MVVWCIKAPAKDRTTRPYHMQLYLEFCKLLFYPFIPEIPFELYLIITEQIWKP<br>GEKILQISVKTIEFGLREAMIAEDEPQRGKAHEQLSDSVQQDIITKANVNIVD<br>MAHANVASLLRTIESLLSSNSPMRSVSCDHREDFSALHEKISSLEVFLKNFEK<br>NNVSRELTDLEVQIKEVANIVEQTIQLRVTEVVLKRHIMVGRDDQKERLLEDL<br>TASYSGEPKVIPIVGMGGIGKTTLAKEVYNNESVLRRFDVHAWATVSQQHDRK<br>EILLGLLRSTIKMEDRVKTRGEAELADMLQKSLKRKRYLIVLDDIWSCEVWDG<br>VRQCFPTEDNAGSRILLTTRNNEVACYAGTENLSMQMDFMDQDESWNLFESAA<br>FSNEASSSEFETIGKKIAEKCHGLPLTIVVVAGLLKSKREIEYWKSVAKDVTS<br>FVTNDPDKQCSRVLGLSYDHLTSDLKTCLLHFGIFPEDSEIPVKKLMRSWMAE<br>GFLKLENDLEGEAEKCLQDLVNRCLVLVCKKSRDETKIISCKVHDLIYELCLR<br>EVERGSVFSMNDIELEVSYSRGLYLSMRKMQPFKHVTGDLSYFGLYRALLTPV<br>HRQLRDHDNNDLSKRTRSIFSFHLKDSSFFLKSELIHFKLLKVLELRHIEIDN<br>FPLQISSLIWLRYLSLVCNANFDVPPEICRLWNLQTFIVQGSQTVITITITIT<br>FPEEIWGLMQLRHLKVKRVYLPNPPSVSADKGSHTRFSNIQTTSYLSSCCCTK<br>EVISGIRNVKKLGFSGNNLSDSGLHSNLVYLQQLEILSFIRCSCILLPVTSAK<br>VFPATLKKLKLESTYLDWSYMDIIAELPNLEVLKLIDDACDGEEWQQNVKGFT<br>RLKLLFIHDNRLKCWKATNDNFPVLERLMLSKCSTLKEMPIEFAEIHSLQLIE<br>LTSCLPGLGKYAARIQKEQQDLGNNPVDVRISNPFTEEVMANKDTELVVTNQI<br>GSSGNENVGDNEEIRKLRRQIIEMHRAWANGLPPPPVPTDNLDYLSSLPPVSH<br>AQLPIFVDMPQHASRSTPGQQYPTTSNIHFLTPQYKITTCSALPAIHAFAAPL<br>PSEAPAFNVNPTVVIPHSTSNPILNIFSDQHYAPKPTFKSTGPYVFPQPPEFP<br>PNTEKPVMTEEQEEIARKLRSLELTMKNLQGLGGYKSVSYKDLCMFPGVHLPL<br>GFKMPKFEKYDGHGDPIAHLRRYCNQLRGAGGKEELLMAYFGESLSGLASEWF<br>VDQDIDKWNSWDDLANEFVQQFQYNMELIPDEKSLTNMNKKSTETFREYAIRW<br>REQAARVKPPMKESKIVEAFIQVQDETYYQHLLPALGKPFVEVPKMGEMIEDG<br>IKTGRIVSFATLKATTQEIHKGLGSMGGRKNMEDASAIAVGQQAWARGPHHRY<br>PRAQTHVYAQAPTNHYQNPLYPVPPPPYQVYNAQPYVQPPSYPHWRAPTLSSH<br>PPTPHTY
778.16	MQSLICDQRVELCALHEKVSSLEVFLKNFEKNNVSGEMTDFEVEVKEVASAAE<br>HAIQLSVTEVVLGENKSQKKKAHRRCHQSLQQVAKDMDRIWTGSTKIQDKGKQ<br>VSKESLVHDFSSSTNDILKVKNNMVGRDDQMKRLLKDLTRSCSDEPKVIPIVG<br>MGGIGKTTLAKEVYNHKPILCHFDVHAWATVSQQHNKKEIFLGLLRSTIKMDD<br>TVKMKSEAELADKLQKSLKRKRYLIVLDDIWSCEVWDGLTRCFPTEDNAGSRI<br>LLTTRNNELACYVDTDNLSLWMNFMDQDESWSLFKSAAFSSEALPYEFQTVGK<br>QSQMNVTEKDLEGEAEKCLQDLVDRCLVLVRMRSRDETKIRSCKVHDLIYDLC<br>LREIQRGNVFTMNDVVFEKPDDCEPAHVSSGCHSLRMALLTPVHRQLRDQDNN<br>DLLKQTRSIFFYDRGISTLILKSELILKSELTHFKLLKVLDVSHVRIDSFPLQ<br>ILSLIWLRYLSLQLLRNLDIPSEICRLWNLQTVIVKGPTGLVITLPEEIWGLM<br>QLRHLKVISSYRQNPLTVSIDKGSRVGSSNIQTTSYLSSCCCTKLVFSRIQNV<br>KKLAFRGNNLSDSGLHNNLVYLQQLEILSFIRCSCILLPVTSAKAFPATLKKL<br>NL

CB.CBv1.2. Scaffold	Amino acid sequence
778.21	MAHACVASLMRTIESLLTSNSPMQSLIYDHREELCALCEKVSSLEVVANNFEK<br>NNVLGEMAELEVEVKEVASAVEHTIQLRVTEVLENDEKSIKIQDKGKQASKES<br>TVQEFPSSPKDILNVENCMVGRDDQRKRLVEDLTRSKSVEPKVIPIIGMGGIA<br>NMLKKSLMGKRYLIVLDDMWKTEAWDAVKLCFPSGNKGSVILLTTRITEVARD<br>AGTKNLSLKTIEELPSDLKTIGKQIAEKCHGLPLTIAVVAGLLKSKRAIADWE<br>NVAKDVKPFVTNDPDKQCSRVLGLSYNHLTSGLKTCLLHFGFFPEDSEIPTKK<br>LMRSWMAEGFLKLENDLE
778.26	MADLEVQIKEVANVVEQTIQLRVTEVVLENDVKAQERLSDSLQQVAEDIDCIW<br>TESTKIQDKGKHASKESTVQEFPSSSRSILNVENSMVGRDDQRKQLVEDLTRS<br>YSGEATVIPIVGMGGNLVEPSVFYKGDKVFTESEAELAYILQKSLKVALYAGT<br>KNSLPMRFMDQDESWNLFKSVAFSSEELPSDLETIGKQIADECHRLWNLQTFI<br>VHGPMLSVITFPEEIWGLMQLRHLKLPRIYLPDCPSGSVDKGRHMDFSNLQTI<br>SYLSSRCCTKEVIMGIQNVKKLRIRRDASSSGPLNNLVHLHQLETLNFTDSLS<br>GLLPASAKAFPATLKKLKLKETGLSWPYLDIIAELPDLEVLKLMDNACLGNEW<br>YPNVRGFTRWKVLLIKRNDLKYWKATDDNFPVLERLMIRSCYYLKEIPIEFAE<br>IHTLQLIELIMCLPELGGFAARIQKEQEELGNNPVDVRISYQWSHSSSFTCYC<br>GFQGDVISLNWKNVAGFVF

Supplementary Table S1. Amino acid sequence of the identified candidate NLR proteins.

Gene	Primer sequence	Purpose
CbAR9	F: AAAAAGCAGGCTAAATGGCAGATGTAGCATTACAACTTGC	Cloning
	R: AGAAAGCTGGGTATCAATGTTTGGGGTTGCAATCAG	Cloning
	R: AGAAAGCTGGGTAATGTTTGGGGTTGCAATCAGC	Cloning
	F: TCTAGAATGGCAGATGTAGCATTACAAC	VIGS
	R: GGTACCTTTGTGATATTTAAGATCCACGAT	VIGS
	F: CCATCCTCTACTACCCACAC	qRT-PCR
	R: TGAAAGTTGTTGAACCCACC	qRT-PCR
778.14	F: CCTGCATTAGGCAAGCCATTTG	qRT-PCR
	R: CTGTTGTCCAACTGCAATGGC	qRT-PCR
778.16	F: GGCTGAGGTACCTATCATTGC	qRT-PCR
	R: TTGAAGAACCCACGCGACTC	qRT-PCR
1022.13	F: ACTCTTCTGATGGGCCTCTC	qRT-PCR
	R: CCGCTATGATGTCCAAGTACG	qRT-PCR
PR1	F: GAGGACAACGTCCGTATGGT	qRT-PCR
	R: AACTCCAGTTACTGCACCATTA	qRT-PCR
PR2	F: CTACTTAAGCTTTGCAAGCACCA	qRT-PCR
	R: AGATCTCTTTCCTCATCGTCACTT	qRT-PCR
ACT	F: GTGCTGAGAGATTCCGTTGC	qRT-PCR
	R: ATGGTTGAGCCACCACTGAG	qRT-PCR
NbPR1	F: GTGCCCAAAATTCTCAACAAGACT	qRT-PCR
	R: AAATCGCCACTTCCCTCAGC	qRT-PCR
NbPR2	F: CAACATAACCTTCCACTCTTAGCCA	qRT-PCR
	R: CATAGAATCCAAAAGGGCATCAAAAAGA	qRT-PCR
NbPR10	F: TGAGATTGTTGAGGGTGATGGGG	qRT-PCR
	R: TTGTCGCCTAAAACATCTCCTTCGA	qRT-PCR
NbACT	F: CCCAGATGGGCAGGTGATCA	qRT-PCR
	R: GAGTTGTATGTGGTCTCGTGGATTC	qRT-PCR

Supplementary Table S2. Sequence of primers used in this study.
